# Fully Integrated Biochip Platforms for Advanced Healthcare

**DOI:** 10.3390/s120811013

**Published:** 2012-08-08

**Authors:** Sandro Carrara, Sara Ghoreishizadeh, Jacopo Olivo, Irene Taurino, Camilla Baj-Rossi, Andrea Cavallini, Maaike Op de Beeck, Catherine Dehollain, Wayne Burleson, Francis Gabriel Moussy, Anthony Guiseppi-Elie, Giovanni De Micheli

**Affiliations:** 1 École Polytechnique Fédérale de Lausanne (EPFL), CH-1015 Lausanne, Switzerland; E-Mails: seyedehsara.ghoreishizadeh@epfl.ch (S.S.G.); jacopo.olivo@epfl.ch (J.O.); irene.taurino@epfl.ch (I.T.); camilla.baj-rossi@epfl.ch (C.B.-R.); andrea.cavallini@epfl.ch (A.C.); catherine.dehollain@epfl.ch (C.D.); giovanni.demicheli@epfl.ch (G.D.M.); 2 Interuniversity Microelectronics Centre (IMEC), B-3001 Leuven, Belgium; E-Mail: opdebeem@imec.be; 3 Department of Electrical and Computer Engineering, University of Massachusetts, Amherst, MA 01003, USA; E-Mail: burleson@ecs.umass.edu; 4 Brunel Institute for Bioengineering, University of Brunel, West London, UB8 3PH, UK; E-Mail: moussyf@who.int; 5 Department of Electrical and Computer Engineering, Center for Bioelectronics, Biosensors and Biochips, Clemson University, Anderson, SC 29625, USA; E-Mail: aguisep@clemson.edu; 6 ABTECH Scientific, Inc., Richmond, VA 23219, USA

**Keywords:** biochip, CMOS design, enzymes, biotechnology, nanotechnology, potentiostats, biocompatible membranes, security, privacy, remote powering

## Abstract

Recent advances in microelectronics and biosensors are enabling developments of innovative biochips for advanced healthcare by providing fully integrated platforms for continuous monitoring of a large set of human disease biomarkers. Continuous monitoring of several human metabolites can be addressed by using fully integrated and minimally invasive devices located in the sub-cutis, typically in the peritoneal region. This extends the techniques of continuous monitoring of glucose currently being pursued with diabetic patients. However, several issues have to be considered in order to succeed in developing fully integrated and minimally invasive implantable devices. These innovative devices require a high-degree of integration, minimal invasive surgery, long-term biocompatibility, security and privacy in data transmission, high reliability, high reproducibility, high specificity, low detection limit and high sensitivity. Recent advances in the field have already proposed possible solutions for several of these issues. The aim of the present paper is to present a broad spectrum of recent results and to propose future directions of development in order to obtain fully implantable systems for the continuous monitoring of the human metabolism in advanced healthcare applications.

## 1. Introduction

According to the Molecular Diagnostics Survey Reports [1], diagnostics testing influences approximately 70% of health care decisions. This means that diagnostics are essential tools for diagnosing and managing numerous health care conditions, ranging from infectious diseases to non-communicable diseases such as diabetes. In fact, non-communicable diseases, or NCDs, are by far the leading cause of death in the world, representing 63% (36 million) of all annual deaths [2].

New diagnostic and monitoring technologies are needed across the economic spectrum, however, since some 80% of all NCD deaths occur in low- and middle-income countries, it is important to address some difficult challenges such as the need for low cost, low power consumption and robustness. Many of these devices should also be usable in places where there are few trained health care workers, and where parts, supplies and maintenance are very difficult to obtain. Fortunately, many of the diagnostics developed for the poorest countries could also have a positive impact in high income countries because their lower cost can help address the rapidly increasing health care costs both for individuals and for national health care systems. Fully integrated biochip platforms could significantly reduce the need for expensive pieces of equipment and laboratory procedures.

Fully integrated biochip platforms can build on existing technologies. Continuous monitoring is already in the market with commercially available devices for glucose [3] and lactate [4] while experimental prototypes have been already proposed for other endogenous metabolites, like glutamate [5] and ATP [6], as well as for exogenous metabolites (typically therapeutic compounds), like cyclophosphamide and naproxen [7]. The technology for continuous monitoring of glucose is robust enough to provide sensors with life-time up to 8 months when implanted in mice [8] and one year in pigs [9]. Sensor performance has been improved to meet sensitivity on physiological concentrations in human blood by using carbon nanotubes in case of both endogenous [10] and exogenous [7] metabolites.

The next step in this development is to hierarchically integrate all of these metabolite sensors in compact and robust biochip platforms to enable minimally invasive human telemetry with negligible costs. Two significant challenges are being addressed: extremely small chip sizes are required to be minimally invasive; and implementation in conventional CMOS manufacturing process is required to assure extremely low per unit fabrication costs. Several additional challenges arise in biosensors, including auxiliary sensors, analog circuit design, remote powering schemes, and security and privacy concerns. Temperature and pH [11] sensors are necessary in the platform for calibration goals. Fully on-board generation of voltage ramps [12] is required to operate the sensing mechanism in the case of drugs [7]. Remote powering is required to address battery-less implants in reducing device size and cost [13]. The personal nature of the data created by biosensors, along with the fact that human health and safety are at stake, requires that security and privacy issues being considered early in the design. Although biosensors can be viewed as just another type of sensor and thus can build on the large literature related to sensor design and sensor networks, the fact that the data is coming from a human being changes the assumptions and threat models. Moreover, the *in vivo* environment presents unique challenges related to calibration, the foreign body response and signalling. These issues will be critical to the acceptance of these technologies by patients, physicians and regulatory bodies, as well as to their responsible deployment in the field.

This paper presents a bottom-up review of some recent advances in these domains and to propose and discuss a comprehensive approach to develop fully integrated biochip platforms for advanced healthcare. Section 2 presents recent advances in using nano-materials (carbon nanotubes, gold nanoparticles, silicon nanowires) to enhance sensor performance in terms of detection limit and sensitivity; Section 3 proposes several proteins (oxidases and cytochromes) used in order to provide specificity to a biochip; Section 4 describes the integration of nano and bio materials onto silicon platforms in order to develop multi-panel arrays; Sections 5 and 6 address the issues related to microelectronics for the biosensing front-end as well as for the problem of remote powering of implanted battery-less devices; Section 7 describes the issues of biocompatibility that are of special importance in developing fully-implantable devices; Section 8 addresses the unique concerns of security and privacy that arise in biosensors that transmit data wirelessly; Section 9 envisages future developments and applications of biochip based platforms connected with geographical networks for fully integrated and advanced healthcare; Section 10 concludes the paper.

## 2. Nano-Materials to Enhance the Sensors Performance

### 2.1. Materials Selection for Advanced Biosensors

Biosensors are based on the principle of converting a biochemical quantity into an electrical signal through the use of electrodes. Currently, a wide variety of different materials are used for the preparation of electrode surfaces for biosensing applications [14]. Most commonly used are glass and other oxide surfaces because of their favourable characteristics [15]. Also widely used are gold [16], micro-porous gold, graphite [17], glassy carbon [18] and indium tin oxide (ITO) [19].

#### 2.1.1. Surface Materials and Modifications

An increasing number of sensing applications use screen-printed electrodes. Screen-printed electrodes (SPEs) are devices that are produced by printing different conductive inks on various types of insulating plastic or ceramic substrates [20]. Graphite materials are preferred due to their simple technological processing and low-cost [21]. Metals such as gold and silver are also used in the construction of SPEs for the analysis and determination of various analytes [22]. In most cases, the working electrode consists of a thin film made by Hg, Au, Ag, Ni or Bi, applied to the graphite surface of the electrode. Renedo *et al.* [8] has reviewed recent developments of screen-printed electrodes and their applications. Another class of materials used for the fabrication of electrochemical biosensors are conductive polymers [23]. Guiseppi-Elie *et al.* has reviewed the use of conductive electroactive polymers in biosensors [24].

#### 2.1.2. Surface Nanostructures

The selection and development of an active material for the electrode is a challenge. The active sensing materials should enable the biological recognition elements (biomolecules, usually proteins or enzymes), to act as a catalyst for sensing a particular analyte or a set of analytes. Recent developments in nanotechnology have revealed several new nano-materials, which have useful properties for numerous electrochemical sensor and biosensor applications [25,26]. By using nanostructures, it is possible to control the fundamental properties of electrode materials and enhance the electron transfer between the electrode and the enzyme, thus improving the catalytic reaction [27,28]. The enhancement of electron transfer is an extremely important challenge in the case of enzymatic biosensors, because a protein shell electrically insulates the redox-active site of most enzymes. Nano-materials such as carbon-nanotubes or nanoparticles have a promotion effect on the direct electron transfer between enzymes and electrode surfaces, thus obviating the need for mediators or co-substrates [29–31].

Many nano-materials (nanotubes, nanowires, nanoparticles, polymers, grapheme, quantum dots), have been used as intermediate layers for integrating electrodes with biomolecules (enzymes, proteins, antibodies, *etc.*), aiming the development of electrochemical sensors for the detection of metabolites and drug compounds [27,28].

### 2.2. Carbon-Nanotubes

**Figure 1.**
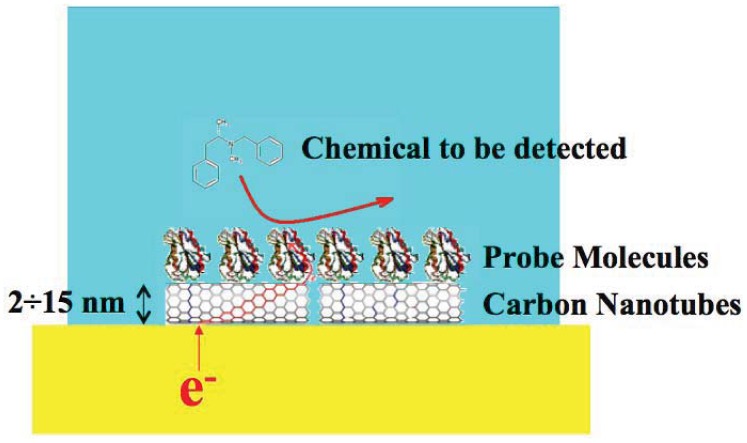
CNT assisted biosensing.

Carbon nanotubes (CNTs) have been recognized as very promising nanomaterials for enhancing electron transfer [32] in biosensing (Figure 1) thanks to their electrical and electrochemical properties which make them suitable to be integrated into biological sensors. For these applications, carbon nanotubes present several advantages: small size with larger surface area, high conductivity, high chemical stability and sensitivity [33], high electrocatalytic effect and a fast electron-transfer rate [34].

Recent studies have demonstrated that CNTs enhance the electrochemical reactivity of proteins or enzymes with retention of their biocatalytic activity [32,35]. The nanotubes and enzyme molecules are of similar dimensions, which facilitate the adsorption of the enzyme without significant loss of its biocatalytic shape, form or function. Carbon nanotubes (CNT) have been extensively studied because of their unique structure-dependent electronic and mechanical properties, which make them suitable for many different electrochemical sensing devices, ranging from amperometric enzyme electrodes [28] to DNA hybridization biosensors [27]. The role of CNTs for the construction of novel biosensing devices has been recently reviewed [27,32,36,37].

In order to integrate biomolecules with CNTs, chemical/electrochemical treatments have to be realized for the introduction of oxygenated functionalities such as hydroxyl groups, which provide sites for covalent linking of biomolecules [27].

There have been a number of approaches to physically adsorb CNTs on electrodes by dispersing in a binder such as Nafion [38], by forming the nanotube equivalent of a carbon paste which can be screen printed, by forming composites with conductive polymers, or by drop casting a solution of CNTs (in solvents such as dimethylformamide [39]) onto an electrode without any binders. Also chitosan has been used for the dispersion and adsorption of CNTs [40–42]. With physical adsorption, the resultant electrode had randomly distributed tubes with no control over the alignment or orientation of the nanotubes. Self-assembly techniques, where aligned nanotubes are directly grown off a surface, were used in order to control the alignment of CNTs [42].

### 2.3. CNT-Hybrid Materials

Hybrid composite materials, based on integration of CNTs with other nano-materials, exhibit special properties due to the synergic effect from the individual components [27]. In particular, due to large improvement of the electron transfer in case of both nanoparticles [43] and nanotubes [44].

Carbon nanotubes–nanoparticle composites have demonstrated an enhancement in the electrocatalytic efficiency of many electrochemical processes [45–47]. Gold nanoparticles [48], metal alloy nanoparticles/multi-walled carbon nanotubes (MWCNTs) [49,50], encapsulated platinum nanoparticles onto MWCNTs [51], and MWCNTs with SnO_2_ nanoparticles [52] are some recent examples.

Carbon nanotube-conducting polymer composites are one of the most widespread approaches for the preparation of electrochemical sensors [53–55]. The combination of the well-known characteristics of conducting polymers (good stability, reproducibility, strong adherence and homogeneity in electrochemical deposition) [56], along with those of CNTs, leads to improve sensing performance, also in presence of biomolecules (e.g., enzyme, proteins). Composites of conducting polymers and CNTs have been synthesized by either chemical or electrochemical polymerization [57–59]. Some recent applications are the incorporation of CNTs in polypyrrole-modified electrodes [60], electro-chemical sensors based on CNT-polyaniline-modified [61], and solubilization of CNTs in poly(vinyl alcohol) (PVA) [62].

Other composites involving carbon nanotubes have been recently developed: a composite film of MWCNTs and cyclodextrin (MWCNTs-CD) as an electrochemical sensor for the determination of adenine and guanine [63], and CNTs and room temperature ionic liquids (RTILs) composites [64]. CNTs incorporated in sol-gel matrices were developed for several electrochemical sensors [65–67].

### 2.4. Nanoparticles

Nanoparticles (normally with dimensions in the range of 1–100 nm) have unique chemical and electronic properties due to their small size that can be used to construct improved electrochemical sensors and biosensors. Different kinds of nanoparticles have been used in different electrochemical sensing systems, such as enzyme sensors, immunosensors and DNA sensors [68]. Generally, metal nanoparticles have excellent conductivity and catalytic properties, which make them suitable for enhance the electron transfer between redox centers in enzymes and electrode surfaces.

**Figure 2.**
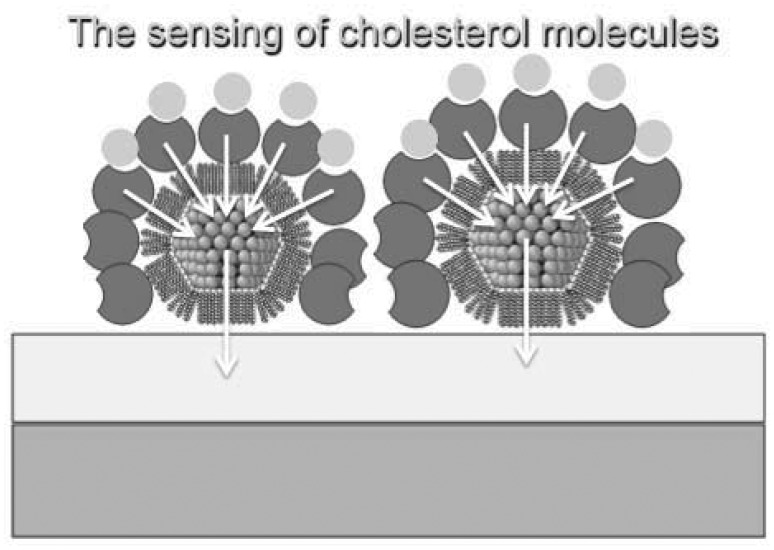
Nanoparticle-mediated sensing.

The main functions of nanoparticles can be summarized as: (1) their ability to facilitate biomolecule immobilization (mainly oxide nanoparticles); (2) catalysis of electrochemical reactions; (3) enhancement of electron transfer through increased surface area (mainly metal-nanoparticles—shown in Figure 2); (4) labelling of biomolecules (quantum dots [69]); and (5) acting as reactant [68,70,71].

Due to their large specific surface area and high surface free energy, nanoparticles can strongly adsorb biomolecules [72]. The adsorbed biomolecules can retain their bioactivity because of the biocompatibility of nanoparticles [73]. Since most of the nanoparticles carry charges and they can electrostatically adsorb biomolecules with different charges. Besides the common electrostatic interaction, some nanoparticles can also immobilize biomolecules by covalent linkage or through the entrapment in polymers. Metal nanoparticles (Au, Ag, Pt), oxide nanoparticles (SiO_2_, TiO_2_, ZrO_2_, MnO_2_, Fe_3_O_4_), or semiconductor nanoparticles (CdS, PbS), have been investigated in recent research and their applications have been newly reviewed [54,55].

### 2.5. Nanowires

Electrochemical sensor devices based on nanowires have been widely reported in the literature [26,74]. Different materials have been investigated for the fabrication of nanowires, such as gold [75], platinum and palladium [76], lanthanide hydroxide nanowires [77], metal-oxide nanowires [78], and silicon nanowires [79].

**Figure 3.**
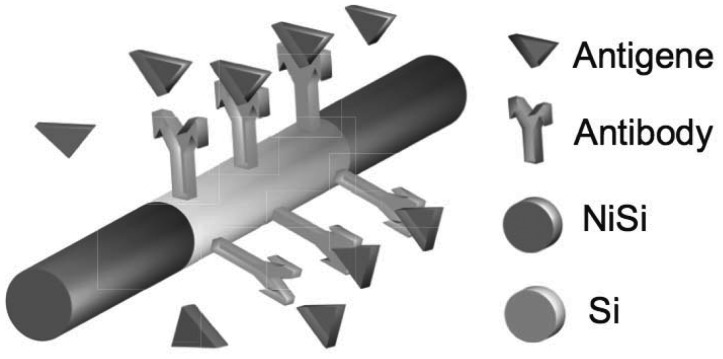
Nanowires based biosensing.

The material properties can be more precisely controlled by manipulating the conditions during nanowire synthesis, using well-developed doping techniques, and by suitable functionalization treatments [74] (e.g., antibodies in Figure 3).

### 2.6. Graphene and Other Carbon-Based Nanomaterials

Another very interesting nano-material for sensors application is graphene. Graphene has shown great promise in many electronics applications because of its unique physiochemical properties: high surface area, excellent thermal and electric conductivity and high mechanical strength [80]. Graphene-based electrodes have shown better enhancement in electrocatalytic activity than carbon nanotubes when used as electrode nanostructures. Several electrochemical sensors based on graphene and graphene composites for bioanalysis and environmental analysis have been developed [81,82]. A new graphene/AuNPs/GOD/chitosan composite-modified electrode was constructed through a simple casting method for a glucose sensor [83]. A single-layer graphene oxide was adsorbed on the 3-aminopropyltriethoxysilane (APTES)-modified conductive electrodes for the fabrication of a glucose sensor based on glucose oxidase [84]. Other carbon-based nanomaterials used for biosensor applications [85] are: (1) carbon nanotube paste electrodes [86]; (2) carbon nanotube nanoelectrode (based on CNT nanoelectrode ensembles) [34]; (3) carbon nanofibers [87]; (4) exfoliated graphite nanoplatelets [88]; and (5) highly ordered mesoporous carbon [89].

### 2.7. Conductive Polymers/Nanocomposites

Nano-structured conducting polymers and polymer composites have recently shown their potential applications in biosensors [90]. Conductive polymer nanowires (CPNWs) are an attractive alternative to silicon nanowires and carbon nanotubes because of their tuneable conductivity, flexibility, chemical diversity, and ease of processing [91]. CP–nanoparticles [92] and CP-carbon nanotubes composite materials [60–62] have also been investigated, due to their hybrid properties.

## 3. Molecular Recognition for Biosensors

The immobilization of a biomaterial onto a conductive support is the basic feature of a biosensor device and also the most critical step. Biomaterials that can be thus assembled include, enzymes [93], receptors [94] antibodies or antigens [95], oligonucleotides or DNA fragments [96,97], or low molecular weight molecules exhibiting affinity interactions with other biomaterials such as cofactors, (e.g., NADPH [98]), or proteins as biotin [99]. Systems that employ enzymes as bioactive interfaces represent the most extensively studied assemblies in biosensors. The high specificity of enzyme-substrate interactions, and the usually high turnover rates of biocatalysts, opens the way to tailor sensitive and specific enzyme-based biosensor devices [100].

Enzymes are proteins that act as powerful catalysts to convert substrates into products [34–40]. Some enzymes, also known as redox-enzymes, catalyse reactions that produce or consume electrons [93]. Direct electrical activation of enzymes, and particularly redox enzymes, represents a general approach to stimulate the oxidation (or reduction) of the enzyme substrates. Electron transfer between the electrode and the redox enzyme should be fast, and the resulting current corresponds to the turnover rate of the electron exchange between the substrate and the enzyme.

Hence, the transduced current (detected with amperometric techniques [14]), reflects the substrate concentration in the system [101]. The functionality of biosensors relies on the electrical contact between enzymes and electrode surfaces. Biomaterials and biomolecules must be assembled on solid conductive supports providing an appropriate electronic communication between the biological matrices and the supporting element [100].

**Figure 4.**
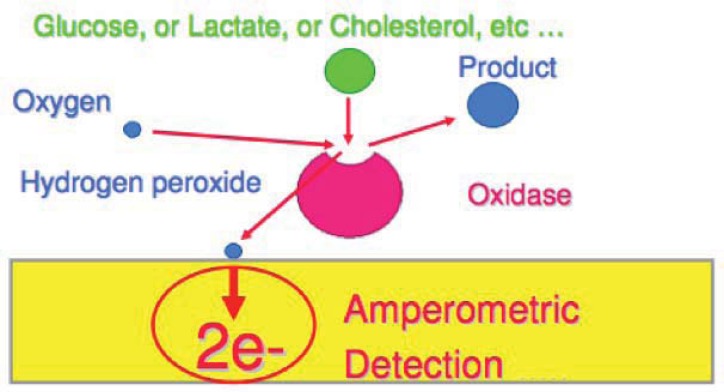
Sensing principle of oxidases.

In general, many oxidoreductases including glucose oxidase, catalyse the oxidation of substrates by electron transfer to oxygen to form hydrogen peroxide [14] (Figure 4). These oxidoreductase enzymes can be immobilized on conducting polymer surfaces and the H_2_O_2_ formed in the reaction is measured amperometrically [57]. The most commonly used enzymes in biosensing are glucose oxidase (GOx) [102] and horseradish peroxidase (HRP) [16]. Other, less commonly used enzymes comprise beta-lactamase [103], and urease [104].

Several studies on amperometric biosensors have been made for the development of drug monitoring-systems, such as the use of the enzyme aryl-acylamidase for assays of paracetamol in connection with the detection of the liberated aminophenol, the use of theophylline oxidase and a ferrocyanide mediator for the biosensing of theophylline, or the immobilization of salicylate hydroxylase for the detection of salicylate in the presence of NADH and dissolved oxygen. Organic-phase tyrosinase and peroxidase electrodes have been used for the monitoring of phenolic and peroxide antiseptics in anti-infective pharmaceutical formulations [105].

**Figure 5.**
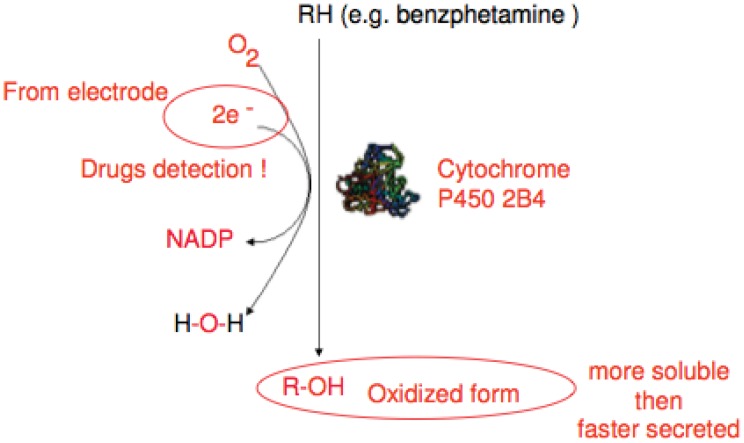
Sensing principle of P450 cytochromes.

Another potential approach is the use of cytochrome P450 enzymes (CYP), as the biological recognition element in amperometric biosensors for drugs monitoring [106–109] (Figure 5). Recently Sharma *et al.* [110], extensively reviewed enzymes used in development of biosensors, also for industrial applications.

Various methods are available for immobilization of enzymes, but not always appropriate for manufacturing biosensors. Several problems relating to the functioning of an enzyme system must be accounted for, like loss of enzyme activity and maintenance of enzyme stability. An efficient enzyme deposition method must satisfy the following requirements [111]:

- The immobilization of the enzyme on transducer surfaces must be stable and provide an efficient electron transfer;
- The enzyme must completely retain its biological activity (and enzyme stability);
- The enzyme must be accessible when immobilized;
- The material used for immobilizing the enzyme must be compatible and chemically inert towards the host environment.

Immobilization of biomolecules can be carried out using many different procedures, like adsorption, entrapment, intermolecular cross-linking and covalent binding [14,110,112,113].

### 3.1. Adsorption

The adsorption process can be classified as physisorption, chemisorption or electrostatic attraction. Binding forces are mainly due to hydrogen bonds, polar and hydrophobic interactions, Van der Waals forces and formation of electron transition complexes [93]. Which intermolecular forces exactly take part in the interaction will depend on the particular protein and surface involved. The resulting layer is likely to be heterogeneous and randomly oriented, since each molecule can form many contacts in different orientations for minimizing repulsive interactions with the substrate and previously adsorbed proteins [96]. However, biosensors with adsorbed enzymes suffer from poor operational and storage stability, so that adsorption in combination with cross-linking has also been used for the immobilization of enzymes [93]. Intermolecular cross-linking of biomolecules are made by reagents such as glutaraldehyde [41], other bi-functional or multi-functional reagents (hexamethylene di-isocyanate, 1,5-difluro-2,4-dinitrobenzene and bisdiazobenzidine-2,20-disulphonic acid, *etc.* [93]) and done in the presence of filler molecules such as bovine serum albumin (BSA) or polyethylene glycol.

Enzymes and nanomaterials are usually dispersed in solvents by sonication, resulting in a suspension. An aliquot is dropped on the electrode surface, followed by air drying, producing the modified electrode. Several solvents can be used for dispersion of nanomaterials and production of electrochemical biosensors, for example dimethylformamide and cyclohexanone [114], chloroform or charged polymers, such as chitosan and nafion. Chitosan is a polysaccharide with good biocompatibility, soluble in slightly acidic solution due to protonation of amino groups and presenting film-forming ability due to insolubility in solution with pH above pKa (6.3). Chitosan has been used in several sensing application as a dispersant agent [115–117]. Electrodeposition of nanostructures and enzyme in chitosan has been successfully performed for the fabrication of sensors as reported in [40,118–120]. Another interesting dispersant is nafion, a perfluorinated sulfonated cation exchanger polymer, showing chemical inertness, thermal stability, mechanical strength, and antifouling property [72]. Nafion is used for the dispersion of nanostructures, in particular, of carbon nanotubes [121–123].

### 3.2. Covalent Bonding and Bioaffinity Immobilization

Modification of electrode surfaces by covalent bonding is advantageous, since it produces a more stable material, resulting in an irreversible binding and a high surface coverage [100], but it requires a high amount of bioreagent and is generally poorly reproducible [93] as such bonding has the potential to adversely affect the active site. Covalent binding is accomplished through functional groups of exposed amino acids of the enzyme, which are not essential for its catalytic activity, such as amino, carboxylic, imidazole, thiol, and hydroxyl groups. These groups create covalent linkage with the functional groups that are available on the surface of the solid support, or that can be generated by chemical or electrochemical treatment. Many procedures have been reported to covalently link an enzyme to a support [100,112].

Immobilization of enzymes can also be achieved by affinity interactions between functional groups such as lectins, (strept)avidin, or metal chelates on an activated electrode surface and an affinity tag such as carbohydrate residue, biotin, histidine or cysteine which is present or genetically engineered by Site Directed Mutagenesis (SDM) at a specific location in the protein sequence [72]. Affinity interaction is stronger than physical adsorption, even if it is not as strong as the covalent linkage, and it provides a basis for a controlled and oriented immobilization of the enzyme on different supports [93,124].

### 3.3. Physical Entrapment

Polymers have gained a remarkable position in the biomedical field as materials for fabrication of various devices and for tissue engineering applications. Polymer films have good stability, reproducibility, more active sites, homogeneity in electrochemical deposition and strong adherence to electrode surfaces [57]. Electropolymerization has received great attention in recent years as a good approach to prepare immobilization matrices for biosensors [56], since it is possible to precisely control film thickness, permeation and charge transport characteristics by the adjustment of the electrochemical parameters. The modification of surfaces with polymeric films has been used in the development of biosensors to protect the surface of the electrodes from impurities, block interfering, incorporating mediators and provide biocompatibility [58].

#### 3.3.1. Conductive Polymers

Conducting electroactive polymers (CEPs) [57,59,111] have attracted much interest as suitable matrices of biomolecules and can be used to enhance enzyme stability and to provide a suitable environment for their immobilization. The techniques of incorporating biomolecules into conducting polymeric films permit the localization of biologically active molecules on electrodes of any size or geometry and are particularly appropriate for the fabrication of multi-analyte amperometric biosensors.

Electrically conducting polymers are known to have considerable flexibility in chemical structures, which can be modified as required. Another advantage offered by conducting polymers is that the electrochemical synthesis allows direct deposition of a polymer on the electrode surface while simultaneously entrapping the protein molecules. Conductive polymers can be reversibly doped and undoped using electrochemical techniques accompanied by significant changes in conductivity. Conducting polymers have the ability to efficiently transfer electric charge produced by a biochemical reaction [57] to an electronic circuit [111].

Conducting polymers belonging to polyenes and polyaromatics such as polyacetylene, polyaniline (PANI), polypyrrole (PPy), polythiophene (PTh), poly (*p*-phenylene), poly(phenylene vinylene) classes have been extensively studied [125]. Recent applications in the field of analytical sensors based on conducting polymer are reviewed by Ahuja [111], Malhotra [57], and Guiseppi-Elie [59].

Biomolecules can be entrapped in conductive polymers through physical adsorption [111,126,127], cross-linking with glutaraldehyde [41], and covalent attachment [128,129], but the most reproducible and controlled technique is the electrochemical polymerization. Entrapment of the enzyme involves electrochemical oxidation of the monomer in the presence of the enzyme to form a polymer, which incorporates the homogenously distributed enzyme molecules during its polymerization reaction. This method is easy to perform and allow a simple one-step fabrication of the electrodes in which enzyme, mediators and additives can be simultaneously deposited simply by adding them to the polymerization solution [111,128].

#### 3.3.2. Enzyme Entrapment/Encapsulation

Enzymes for biosensors have been entrapped in membranes based on advanced materials, including hydrogels (which contain cryohydrogel, organohydrogel and grafting copolymer), sol-gel-derived organic-inorganic composites, and lipid membranes [130].

Hydrogel, based on a cross-linked network of polymers able to swell with water, is one of the ideal enzyme-immobilization materials, providing a biocompatible microenvironment for the enzyme to maintain its natural configuration [131], and at the same time mediating electron transfer between the enzymes and the electrode via electron diffusion through mobile polymer segments [14]. The most used hydrogels for immobilization of enzymes are poly(vinyl alcohol) (PVA) [132], or PEG (polyethylene glycol), which prevents nonspecific adsorption of biomolecules [133]. A kind of polyhydroxycellulose (PHC), a mixture of PVA and carboxymethylhydroxyethyl cellulose, was investigated as a novel immobilization method for enzymes [130].

Sol-gel processes form sol-gel-derived organic-inorganic composites. They involve hydrolysis and co-condensation of silane precursors like tetramethoxysilane (TMOS) and tetraethylorthosilicate (TEOS), with acid or base catalysis. The material formed provides a promising means for immobilizing bioactive molecules [134]. Further, addition to the starting sol of polymers, e.g., poly-dimethyl-siloxane, polyamides, polyacrylates, polyethylene glycol (PEG), and poly(ethylene-oxide), was investigated to regulate the inorganic condensation–polymerization process [130]. Polyaniline (PANI) was electrochemically entrapped onto TEOS-derived films on indium tin oxide (ITO) coated glass to immobilize lactate dehydrogenase (LDH) for lactate sensing [135]. Acetylcholinesterase (AchEs) enzyme in TMOS+MTMOS+PEG-derived sol-gel has been immobilized on a modified screen printed electrode for the detection of carbamate insecticides [136].

Lipid membranes, imitating the basic structure of nature's biomembrane, represent a relatively biocompatible structure for the development of new types of electrochemical sensors [130]. Lipid membranes can be formed by casting solutions of lipids or surfactants in organic solvents or of aqueous vesicle dispersions onto solid surfaces. Recently tethered bilayer lipid membranes (tBLM) have been developed. In tBLMs, the bilayer is attached to the surface via special chemical anchors, which on one side are bound to the surface and on the other side insert into a bilayer leaflet. For this purpose, lipid derivatives (phospholipids, cholesterols, alkyl chains or phytanyl groups), have been synthesized, which, via a hydrophilic linker, are connected to a group that binds strongly to the surface [137,138].

Dialysis (ultrafiltration) membranes have also been used [139], to entrap the enzyme between the electrode and a dialysis membrane having a cut-off inferior to the size of the enzyme, while the substrate is able to freely diffuse through the dialysis membrane. Such an immobilization method allows small amounts of enzyme to be used and the integrity of the enzyme to be preserved [93]. Immobilization layers based on clay-materials were also investigated in some sensing applications [140].

### 3.4. Langmuir-Blodgett, Layer-by-Layer, Self-Assembled-Monolayer (SAM)

Two of the most used methods for the immobilization of biomolecules on electrodes are the electrostatic layer-by-layer (LbL) and the Langmuir-Blodgett techniques, which are complementary to each other in terms of the types of material that can be employed.

#### 3.4.1. Langmuir-Blodgett Based Electrochemical Biosensors

The Langmuir-Blodgett (LB) technique promotes a high control of the physical and chemical properties of nanostructured organic films and it has been widely used for the production of miniaturized devices for enzyme immobilization. The LB technique is based on the transfer of insoluble monolayers supported on liquid-air interfaces to solid supports that intercept vertically the liquid surface. The material can form a monomolecular film at the liquid-air interface in such a way that hydrophobic moieties are oriented towards the air and the hydrophilic ones towards the aqueous phase. This film, usually called Langmuir film, can be compressed through liquid barriers in order to obtain the desired surface density [72].

The LB technique is a suitable method for immobilization of enzymes because of its well-ordered structure of the ultrathin films and its ability to control the amount of biomolecules immobilized by changing the number of deposited layers. The classical materials used to form Langmuir and LB films are lipids, such as fatty acids, glycerophospholipids, ionic liquids [141], synthetic polymers [142], proteins [143], nucleic acids [144], and polysaccharides [145]. Girard-Egrot recently reviewed applications of enzyme with lipidic Langmuir–Blodgett films [146].

Electrochemical sensors based on the LB technique contain enzymes such as horseradish peroxidase [147], hemoblogin [148], glucose oxidase [149], acetylcholinesferase [150], urease [151], tyrosinase [152], choline oxidase [153], lactase and galactose oxidase [154], uricase [155], for detection of several substances including hydrogen peroxide, glucose, choline, urea, phenols, lactose and uric acid.

#### 3.4.2. Layer-by-Layer (LbL) Based Electrochemical Biosensor

The LbL technique has been found to be a useful way for assembling a number of organic and inorganic substances, including proteins, DNA, viruses, dendrimers, and nanoparticles [156]. Basically, the processes of film fabrication by the LBL technique are governed by the adsorption of organic polyelectrolytes (PE) with opposite charges present on their molecular structure, in such a way that film thickness, porosity and morphology can be precisely controlled according to the application to a target. An important advantage in the use of the LbL technique to construct biosensors is the possibility to incorporate inorganic materials and biomolecules [157].

Multilayer films are grown by alternate dipping of the modified electrodes into the enzyme solution (or nanoparticles solution) and a PE solution, respectively. Polycations that have been used in LbL films formation include poly(allylamine) (PAA), poly(L-lysine) (PLL), poly(ethyleneimine) (PEI), poly(dimethyldiallylammonium chloride) (PDDA), poly(allylamine hydrocholoride) (PAH) and chitosan (CHIT). The most commonly used polyanions are poly(stryrenesulfonate) (PSS), poly(vinylsulfonate) (PVS), poly(anilinepropanesulfonic acid) (PAPSA), poly(acrylic acid) (PAA) and poly (methacylic acid) (PMA) [157,158]. Water-soluble proteins such as cytochrome c (Cyt) [159], horseradish peroxidase (HRP) [160], myoglobin (Mb), lysozyme (Lys), histone f3, hemoglobin (Hb), glucoamylase (GA), and glucose oxidase (GOx) have been incorporated in LbL and used for biosensors [158,161].

However, in the development of electrochemical biosensors based on LBL assembly methods, other strategies such as biological affinity, covalent binding, cross-linking, and electrodeposition have also been used, and also multi-strategies have been investigated [158].

#### 3.4.3. Self-Assembled-Monolayer (SAM)

Redox proteins can be adsorbed via electrostatic interaction or covalently immobilized on Self-Assembled-Monolayers (SAM) [162], which can lead to a better-controlled electron transfer between the protein and the electrode [163]. With SAMs it is possible to regulate the distance between the enzyme binding-site and the electrode surface, changing the chain length (the so called ‘tail’), of SAM, which allows the film to be tightly packed and oriented on the surface thanks to the weak interactions between the tails. Usually alkane-thiol or other thiol-terminated chains covalently bind the self-assembled molecule to the metal surface of the electrode. At the other end of the chains, a group is able to specifically interact with a group on the protein surface, thus adding selectivity to the modified electrode for particular proteins.

## 4. Integration of Bio and Nano Materials on Multi-Electrode Platforms

**Figure 6.**
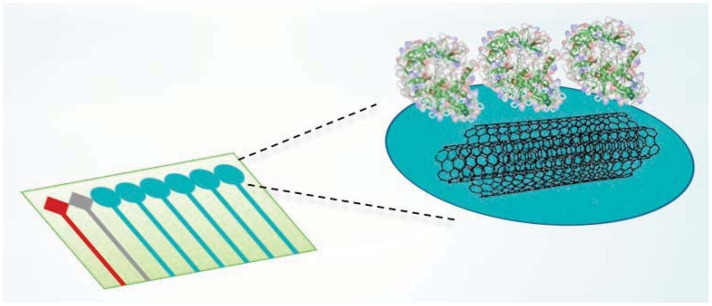
Multi-array platforms and CNT integration.

In the last few years the use of miniaturized electrodes fabricated by thin-film technology has become more and more common in sensor research because it gives some advantages (e.g., smaller background current and faster time response). The combination of microelectrode devices with the immobilization of biochemical compounds such as enzymes provides excellent prerequisites for the development of miniaturized biosensors in order to monitor many metabolites in parallel [164]. These biodevices should be composed of working electrodes that share the same counter and reference electrodes. Working electrodes have to be functionalized first with MWCNTs to increase sensitivity and decrease the detection limit (Figure 6).

**Figure 7.**
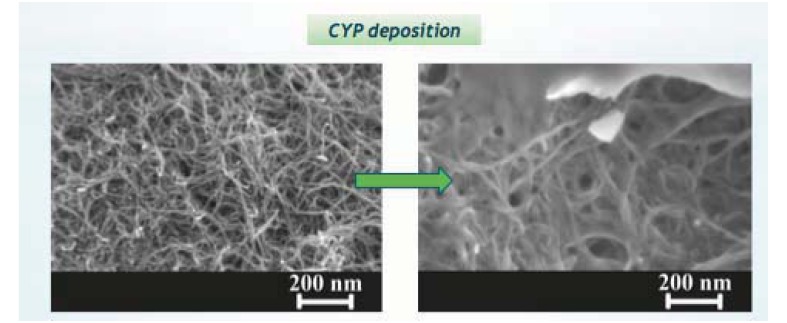
Enzyme incorporation onto CNTs.

Then, the enzymes have to be incorporated to enhance the selectivity for each metabolite (Figure 7). A fixed or variable potential between a reference electrode and working electrodes must be applied according to the functionalization of each electrode.

### 4.1. MWCNTs Integration Methods

#### 4.1.1. Direct Growth onto Chip

**Figure 8.**
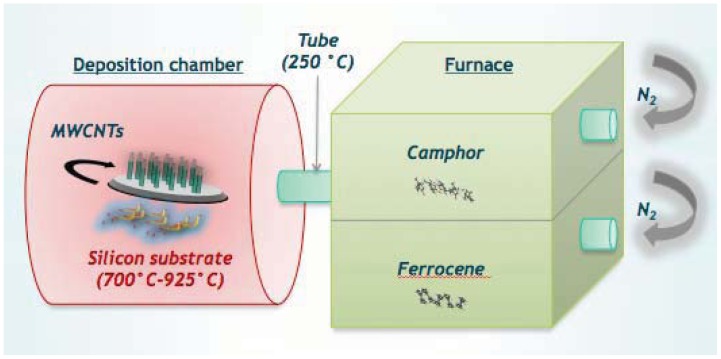
CVD growth of CNTs.

Direct growth by Chemical Vapour Deposition (CVD), is one of the most used forms of growing CNTs. In CVD, a hydrocarbon gas with carbon atoms is provided for the CNT growth on metallic catalysts [165]. The catalyst can be introduced in a vapour phase or is present as a solid layer on the substrate on which CNTs have to grow (Figure 8). Typically, nanotubes are grown on semiconducting or usually insulating silicon, silicon dioxide, quartz. The whole setup is then placed in a quartz tube, maintained at atmospheric pressure in a flow furnace. The hydrocarbon gas mixture is passed over the quartz at high temperatures (500–1,000 °C). The gas catalytically decomposes over the metal particles (iron or nickel) at these temperatures. Compared with other methods of CNT production, CVD has been regarded as the most promising for industrial application. In particular, CVD is the preferred choice to grow patterned CNTs on metal catalyst particles or islands deposited on top of the substrates. These catalytic metals are seeded onto metallic tracks realized on a substrate through standard microfabrication techniques (standard UV photolithography, electron-beam evaporation, lift-off).

For many applications, such as biosensing, high electrical conductivity is required usually needing a conductive substrate to connect with CNTs. It is challenging to grow CNTs on metal substrates since the elevated temperature may activate the diffusion of the catalyst material into the substrate, which inhibits its activity. Catalysts tend to form alloys with substrates or to coarsen and segregate during growth [166,167].

To overcome this problem, one possible strategy is to employ pure catalytic metal [168] or alloys [169] containing at least one of these well known catalyst materials as substrates. However, if the substrate selection is constrained to specific metals an alternative effective choice is to deposit a thin insulating layer to stop the poisoning of catalyst particles. Insulating layers, however, cannot provide an electrical connection to the conducting substrate, and additional fabrication processes are required to electrically contact with the substrate [166]. Nevertheless, some researchers succeeded in growing CNTs directly on metal substrates by using vapour pressure catalyst delivery [170]. This technique cannot be applied to the patterned growth approaches needed for biosensing applications.

It is a further challenge to grow CNTs on metallic substrates at CMOS compatible temperatures (400–450 °C) [171]. Lowering the growth temperature has frequently proven to be ineffective because the structural quality of CNTs usually degrades as reaction temperatures are decreased. Moreover, higher temperatures are often required to form the metal nanoparticles necessary for CNT growth.

Initial approaches to overcome these drawbacks consist of improving the CVD recipes by varying the reactant gases, pre-treatment and immobilization method of the catalyst and other deposition parameters. Up to now only few papers have reported this kind of approach for the realization of enzyme biosensors. Lin *et al.* [172] reported a glucose biosensor based on CNT nano-electrode ensembles made of low-site density aligned CNTs grown on a chromium-coated silicon substrate by plasma-enhanced CVD and using nickel nanoparticles as a catalyst. An epoxy-based polymer was then spin-coated and the protruding parts of the CNTs were removed by polishing. Then, glucose oxidase was immobilized directly on the broken tips of CNTs for glucose detection. In addition, Tominaga *et al.* [173] studied the direct electron transfer reactions of D-fructose dehydrogenase immobilized onto MWCNTs for D-fructose detection. The CNTs were synthesized by CVD method using iron nanoparticles derived from ferritin molecules and adsorbed onto the platinum plate.

CVD incorporation of CNTs not only improves the electrical contact between the active sensing material and the conducting substrate, but also ensures that the sensor is free of any other materials used as surfactants and/or binders [174]. However, due to the CNT hydrophobicity, CVD-CNTs need to be chemically and/or electrochemically treated for the easy incorporation of the enzymes. Finally, CNTs grown by CVD may collapse in the presence of solvents.

#### 4.1.2. Covalent Bonding

The advantage of covalent bonding is linked to the great chemical affinity between gold and thiol and the good stability of the Au–S bond. Common to these methods is the formation of carboxylic acid groups on the surface of the CNTs, usually introduced by strong acid oxidation. They occur predominantly at the more reactive (open) ends or at defect sites of CNTs. Exploiting the high chemical reactivity of this functional group, it is possible to covalently attach biomolecules obtaining in this case a stable grafted surface that could be particularly attractive for biosensing purposes [175].

Typical approaches reported in the literature to link covalently CNTs onto gold include Au-S bonding and surface condensation. The first strategy mimics the formation of alkanethiol SAMs on a gold surface. Carboxyl-terminated MWCNTs must be modified with 2-aminoethanethiol and vertically self-assembled on gold electrodes. Substrate dipping in a suspension of functionalized CNTs such as ethanol [173], chitosan and tween [176] allows them to be vertically assembled onto the gold surface. With the latter method, the carboxylic functionalized MWCNTs are usually dissolved in an organic solvent and the gold substrates must be pre-modified with amino groups [177]. The surface condensation reaction is carried out with 1-ethyl-3-(3-dimethylaminopropyl)carbodiimide hydrochloride (EDC) as coupling agent.

In a mixed procedure, Kim *et al.* [178] added alkanedithiols to the surfaces of MWCNTs and immobilized them on SAM-modified Au substrates by dipping these substrates into DMSO solution containing MWCNTs. The adsorption of MWCNT-SH onto SAMs of alkanethiols takes place selectively depending on the chain lengths of the thiolate in the SAM.

One limitation of such integration is that the highly conductive CNTs were not in direct contact with the electrode surface and thus electron transfer could be hindered. In addition, different methods to link covalently SWCNTs-Au have been well established [179] especially for biodetection. However, to the best of our knowledge, these studies have not been published for MWCNTs despite their better electron conduction properties. It is due to the following challenges:

- MWCNTs are more difficult to disaggregate and suspend in solvents than SWCNTs.
- The common procedure employed to introduce thiol moieties selectively on the tips of the SWCNTs also modifies the sidewalls of MWCNTs inducing in most cases their random orientation onto the gold electrodes.

#### 4.1.3. Micro-Spotting

**Figure 9.**
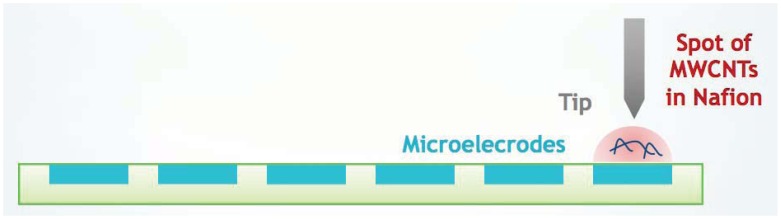
Microspotting.

Micro-spotting casting immobilization is the most widely used method for fabricating CNT-based electrochemical sensors (Figure 9). With this approach, MWCNTs must be dispersed in a certain solvent with sonication after their purification and activation pre-treatments. The resultant suspension must be cast onto the electrodes and allowed to dry. In addition to different solvents such as chloroform [10], various additives are added into solvents to assist the CNT dispersion (e.g., Nafion, polymers) [174]. The predominance of these methods is their simplicity, which makes them suitable to fabricate CNT-based electrochemical sensors.

A microspotting system was developed for the rapid assembly of MWCNTs onto small surfaces [180–182]. In general, a micro-robotic station included three programmable components [180]:

1. a computer controllable X-Y-Z micromanipulator of the probe tips to appropriate location of them;
2. a computer controllable hydraulic pump system to inject CNT dilution through the capillary probes; and
3. a camera allowing an operator to locate microelectrodes.

The CNT suspension is transferred to the probe tip by the micro-pump. The probe is then commanded and moved to the desired position by using the computer controllable micromanipulator automatically. Finally, the CNT suspension is deposited onto the desired location [180].

CNT dilution spotting is challenging because of the difficulties in casting a very small CNT-based solution to particular positions automatically and precisely [181,179]. This technique was employed by Lai *et al.* to connect CNTs across two microelectrodes. CNTs were suspended in acetone [182] or ethanol [179,183] solutions. It was the first research group that employed and optimized the micro-spotting deposition process of CNTs by comparing the spot sizes, by using different probes and by studying the micromanipulator options to change the probe positions [179,183]. They tested also two different spotting methods to study the performance of the micro-robotic spotting system: injection method, spotting using a syringe pump to induce enough hydraulic pressure and droplet contact method, spotting by allowing a droplet to come in contact with substrate [180]. In the biosensing field, Boero *et al.* used a MWCNT solution based on Nafion. It was preferred to dispersion in chloroform since the last one is too volatile for the small volumes involved (400 pL). They performed automatic spotting of MWCNTs with a commercially available non-contact spotter. Multiple layers were spotted allowing Nafion solutions to dry in between two subsequent depositions [181].

## 5. CMOS Design for Highly Integrated Potentiostats

### 5.1. Circuit Design for Implantable Biosensor

**Figure 10.**
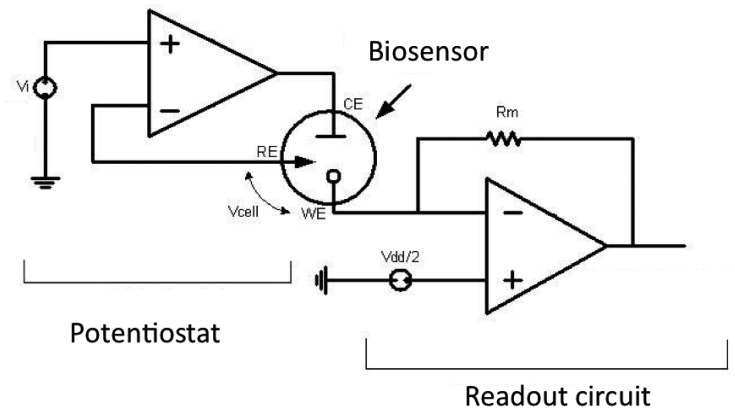
Simplified frontend electronics.

To realize fully implantable and minimally invasive biosensor platforms, the sensor and the front-end electronics should be miniaturized and integrated into a single chip. Thus low power and low noise analog/mixed-signal integrated circuits must be carefully designed for all different types of measurement procedures. The required circuit elements to implement the front-end electronics can be divided into two main parts:

1. readout circuit;
2. voltage generator and potentiostat (Figure 10).

These parts will be introduced in the following subsections. The biosensor has 3 electrodes: working electrode (WE) reference electrode (RE) and counter electrode (CE).

#### 5.1.1. Readout Circuit

The readout circuit serves to detect and amplify the sensor signal and convert it to a form which is can be modulated or transmitted. The readout circuit may include current mirrors, current amplifier, operational transimpedance amplifier (OTA), analog to digital converter (ADC), current peak detector, *etc.* As the miniaturized sensor has a very weak signal (pico- or pA), the readout circuit must be designed to have low noise and low leakage. On the other hand, as the whole system will be remotely powered, it has to consume low power, on the order of a few microwatts.

**Figure 11.**
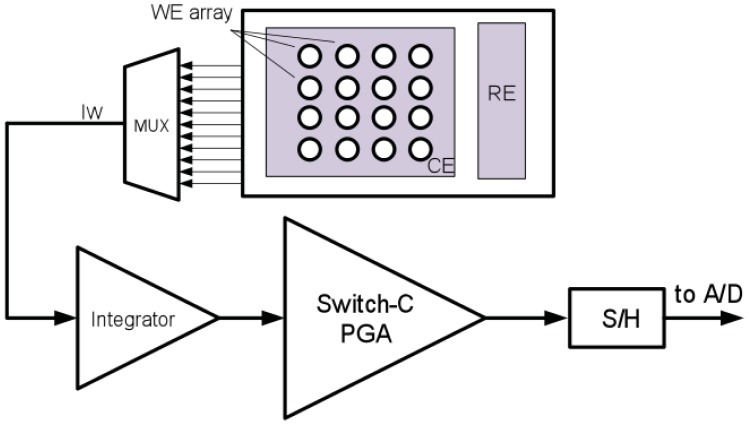
Schematic of the surface electrode array with the potentiostat and the readout circuit.

Many works have been done to design biosensor readout circuits. In the work of Ahmadi and Jullien [184] and Haider *et al.* [185], the sensor current was converted to frequency, which can be sent out by backscattering without any need for an ADC. However this complicates data processing steps such as error correction, storage or data encryption. In Li *et al.* [186] a switch-capacitor was used together with a synthesis method to suppress the noise and the offset of the amplifier. It amplifies a wide range of sensor current 100 fA–100 pA and converts it to voltage. Then, using an ADC, the voltage was digitized and can be modulated and sent out of the implanted device (Figure 11).

Another method that is introduced in [187] is to amplify the sensor current and digitize it using a current mode ADC [187] and [188] without converting it into voltage. This method doesn't need an OTA. The other advantage of the method is that the digitized signal can be processed and encrypted before being sent out. In yet another approach [189] used a low pass filter and an ADC to digitize the potential decay on a capacitor produced by the cell current.

#### 5.1.2. Potentiostat and Voltage Generator

A potentiostat circuit has the purpose of keeping the reference and working electrode at a given potential to provide a stable bias voltage for measurement purposes. The stability of the potentiostat circuit should be studied carefully as the electrical model (*i.e.*, the RC equivalent model) of the biosensor can change significantly with the surrounding fluid concentration, temperature and also the applied voltage to the sensor. Therefore, to prevent any instability, a safety range in the phase margin of the amplifiers should be applied when designing the potentiostat, so that it does not become saturated by changes in the properties of the surrounding fluid (concentration, density, temperature, *etc.*) within the pharmacological range.

Another concern in designing the potentiostat is that the CE (Counter Electrode) voltage depends on the type of the sensor, cell current and the fluid properties. Thus, if the potentiostat limits the range of the CE voltage, it can potentially affect the accuracy of the measurements. Therefore, the potentiostat should be designed in such a way to let the CE change freely within the voltage range. This range can be acquired by modelling of the biosensor, or with some experiments prior to connecting the biosensor to the electronics.

**Figure 12.**
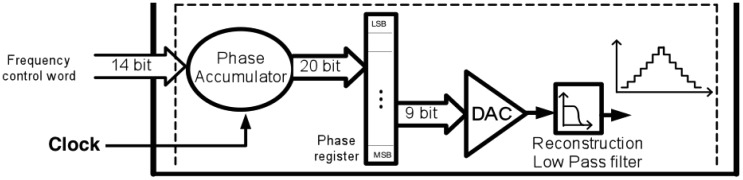
Direct digital synthesizer (DDS) to generate a very slow ramp to drive a cyclic voltammetry measurement.

The potentiostat can be designed using one of many amplifiers in different configurations [190,191]. Depending on the type of the measurement, a fixed or a variable voltage is needed to apply to the cell. For single-target chronoamperometry, the voltage is fixed and chosen on the basis of the electrochemical reaction. For cyclic voltammetry, this circuit sweeps repeatedly within the voltage range of interest. A band-gap reference circuit can be used to generate a fixed voltage [192]. To design a very slow triangular waveform voltage, a mixed signal design approach can be used (Figure 12). In [186], a digitally programmable waveform generator was used by employing an up-down counter, comparator, latch and a digital to analog converter (DAC).

### 5.2. Multi-Target Sensing

The integration of multiple biotransducers into sensors [193] with the required electronics can pave the way to use smart systems in clinical diagnoses [194,195]. Therefore multiplexing circuits are required to support the readout of multiple current sources and the drive of multiple control points for the potential. Multiplexing can also be used to send out other data from auxiliary sensors such as the temperature and PH of the fluid as introduced in [11].

### 5.3. Different Noise Sources

The main sources of noise that can affect the system performance can be classified into three categories: (1) potentiostat and voltage generator noise; (2) current readout and amplification circuit; and (3) biochemical environment. The noise performance for the potentiostat and voltage generator block is not critical because the stimulus signal for amperometric interrogation is normally several hundred mill-volts, well above the noise level. However, in the current readout block, noise performance will significantly affect readout sensitivity because the response current of the biosensor could be very small (in sub-picoamp range).

**Figure 13.**
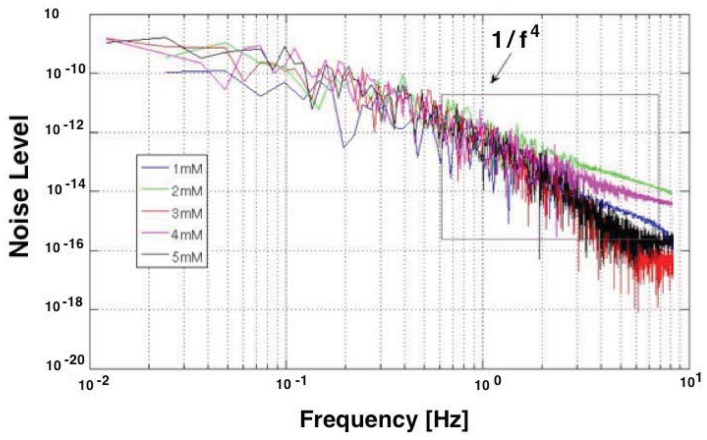
Noise PSD measured with screen-printed electrode at V_cell_ = 650 mV.

The electrochemical noise includes the noise generated in the interface of the electrode, carbon nanotube, protein (enzyme) and the substrate. The low frequency component of the biochemical environment noise limits the sensitivity and determines the detection limit of the biosensor. So the noise from the biochemical environment should be minimized to enhance the biosensor performance. This is not straightforward, since the sensor noise is hard to quantify analytically, but it can be measured experimentally [164]. Figure 13 reports the Power Spectrum Density (PSD) registered with a sensor.

It is an example of the measured low frequency noise of the biosensor during the detection of H_2_O_2_. Some research has been done to model the noise of the carbon nanotube devices [196], neural sensors [197], and electrode-electrolyte interface [198]. However a closed form model for the biosensor noise has not yet been developed.

## 6. Remote-Powering

In realizing implantable biochip platforms, providing energy to the implant becomes a relevant issue if needed to decrease the impact on the patient in terms of pain and device size. Energy harvesting techniques might exploit natural or artificial power sources surrounding the person to provide energy to implanted circuits. The energy can be used directly or stored in capacitors or batteries [13,199]. Thus, batteries can either be eliminated or be smaller and with a longer lifetime. However, batteries may be avoided in order to decrease the biochip size with benefit on the patient's side. Remote powering is one of the best approaches if we would like to avoid batteries. In remote powering, an alternating current flows into an inductor in an external base station; and the resulting variable magnetic field generated induces an alternating current into one or more implanted inductors. In such cases, the system works in the near field region [200,201] where the distance between the external base station and the implant is smaller than the wavelength divided by two times pi (λ/2π) [202]. It exists in typical frequency bands for such applications, which are centered at 125 kHz, 6.7 MHz or 13.5 MHz [202].

The techniques of near field communications (NFC) and powering have been studied for several decades, and has been rapidly appearing in the marketplace for RFID tagging, mobile payments and other applications [203]. However, the miniaturization process of the implanted inductors to the millimetre level, while preserving the power efficiency, is still an open research topic. Moreover, it is not an easy task to maximize the total power efficiency of the remote powering link from the external base station to the output of the AC to DC converter, also called rectifier, as shown in [201]. This is due to the fact that this total power efficiency depends on several different blocks such as the Power Amplifier (PA) of the base station (in most cases a class E PA), the design of the primary coil of the base station, and of the secondary coil of the implant, the topology of the AC to DC converter, the voltage regulator, and others.

**Figure 14.**
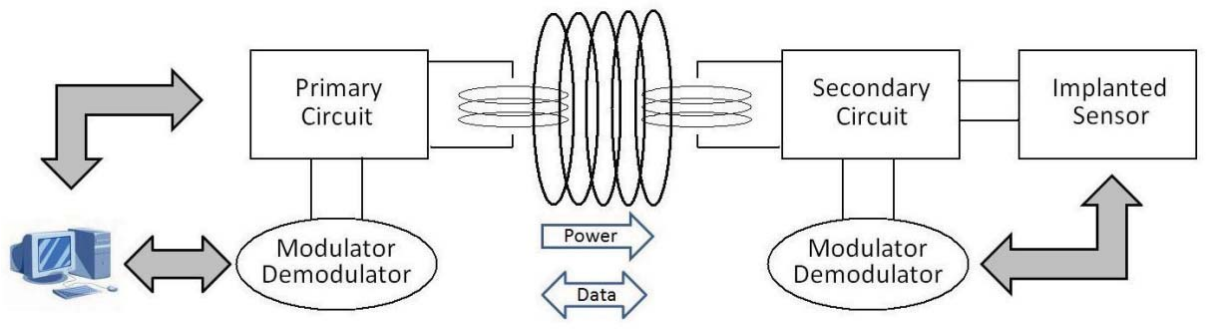
Schematic representation of an inductive powering performing bidirectional data transmission.

Inductive links present considerable additional complexity, when compared with other kinds of power transmission. Exploiting this technique, data can be transmitted from outside to inside the body (downlink) and *vice versa* (uplink) (Figure 14) without using a radio-frequency active transmitter which would otherwise consume significant power due to the RF oscillator and amplifier. This can be feasible by instead modulating the load of the secondary coil, varying in this way the total load seen by the primary coil. This technique of data transmission, often named load-modulation, allows significant energy savings by avoiding the use of an implanted RF active transmitter. Indeed, the RF active transmitter would otherwise have the highest power consumption among the components of an implantable biosensor. The capability to avoid an implanted RF active transmitter, together with a delivered power of up to a few milliwatts, makes this technique particularly suitable for very small (*i.e.*, minimally-invasive) implantable biosensors.

**Figure 15.**
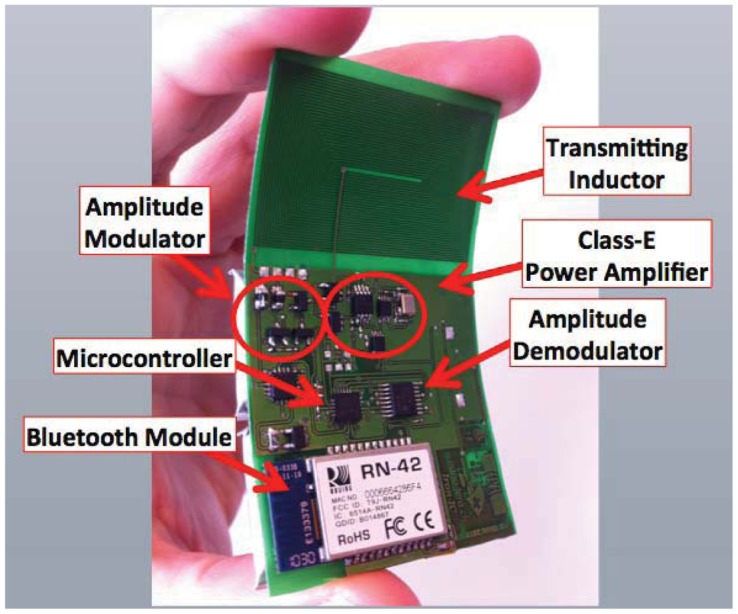
Photo of the prototype IronIC Patch. The system can be placed over the implantation area with an adhesive bandage to power and communicate with implanted sensors.

A wearable device to transmit power to an implantable sensor has been recently presented [204]. The prototype system, named IronIC Patch, is shown in Figure 15. The system is realized on a flexible FR4 substrate to be embedded into an adhesive skin patch and located directly over the implantation area. This placement reduces the probability of misalignments between the transmitting and receiving inductors, thus significantly improving the efficiency of the link for both power and data transmission.

The IronIC Patch can transmit power wirelessly to the implanted sensors by driving the transmitting coil with a class-E power amplifier. Downlink communication is achieved by means of an Amplitude Shift Keying (ASK) modulator. Uplink communication is achieved by monitoring the current drawn by the amplifier. The system also enables long-range communication with more powerful remote devices (e.g., mobile phone) by means of an embedded bluetooth module. Finally, the device is powered by two rechargeable lithium-ion polymer batteries. The system has been demonstrated to be particularly suitable to power implantable biosensors dedicated to glucose and lactate monitoring [184,205].

## 7. Biocompatibility

Biocompatibility is a critical issue that must be solved in order to implant electronics and sensors into the human body. Biocompatibility aspects cannot be neglected otherwise the implantation can cause important adverse effects to the body such as severe inflammation and necrosis of the tissue surrounding the implant in the short term, as well as long term systemic effects such as allergic reactions or even the onset of cancer. Furthermore, if a pronounced mismatch exists between the local body tissue and the implant, a pronounced ‘Foreign Body Response’ (FBR) will occur, causing walling of the implant by fibrosis after 3–4 weeks of implantation [206]. Due to the presence of this fibrotic tissue, chances are high that the sensor is not in contact with representative tissue/body fluids, causing an incorrect readout of the sensor. The preferential adhesion of certain proteins or cells onto the sensor, occurring very fast after implantation, might result in incorrect readings too. A second important aspect for all active implants is their biostability: an implant should remain functional after implantation; hence no body fluids should penetrate into the device causing corrosion and disabling the correct functionality of the device. In this section we will present an overview of the foreign body response to an implant, and strategies aimed to solve specific biocompatibility and biostability issues.

### 7.1. Biocompatibility

Biocompatibility has been defined as the ability of a material to perform with an appropriate host response in a specific application [207,208]. An appropriate host reaction includes no local tissue damage due to cytotoxicity, limited FBR and no short or long term adverse body effects. Also, this definition points out that a precise metric of biocompatibility does not exist. The site and duration of the implant, as well as type of material-body contact are important parameters, which makes biocompatibility a contextual concept. Even more, the same material can induce adverse or favourable biological response according to its particular employ [209].

Experiments to test biocompatibility are carried both *in vitro* and *in vivo*, in order to test the local and systemic effect of the materials. The ISO 10993 standard [210] is a well-known and constantly updated standard for evaluation of biocompatibility prior to clinical testing of a device. Various tests are essential for each material/device before medical use. The three most basic evaluations are cytotoxicity, sensitization, and irritation testing. The presence of toxic, leachable substances is investigated, causing adverse effects on biosystems. Based upon the final goal of an implant (implant duration, location in the body, and type of tissue contact), various additional categories of tests have to be performed, such as genotoxicity, hemocompatibility, carcinogenicity tests, *etc.* Obviously, a medical glove for short time contact with the skin will need much less testing compared to a heart assist device which will be implanted in the body for over 10 years.

### 7.2. The Foreign Body Response

In order to better understand how biocompatibility can be improved, it is useful to know how the body reacts to an foreign implant. A detailed description of this reaction can be found in [207,208]. Below a summary is given.

As introduced above, the natural response of the host to the introduction of exogenous material is called the *‘foreign body response’* (FBR). Immediately after implantation in the body, the implant spontaneously acquires a layer of host proteins. Composition, concentration and conformation of this protein layer is dependent on the foreign material and the implantation site. As a consequence of the insertion, a fibrin clot is formed to close the lesion, while an oedema is formed close to the wound. Neutrophils and macrophages present at the injured site start to clean up the damaged area. These leukocytes will release molecules, to signal to the immune system that a wound exists and to promote further infiltration of white cells at the injured site. In this phase, the host experiences a period of acute inflammation—the injured site will swell and is warm and painful.

Persistent inflammatory stimuli given by various leukocytes will lead to chronic inflammation. This stage is characterized by the presence of monocytes, macrophages, lymphocytes and plasma cells at the implant site, and a proliferation of blood vessels and connective tissue to heal the affected area. The macrophages will clean up the wound site, but they will also try to digest the foreign material. When the individual macrophages can't digest the implanted material/device, the macrophages form clusters called ‘foreign body giant cells’ in a final attempt to digest the foreign material. When using biocompatible materials, usually chronic inflammation last no longer than 2–3 weeks. In the final stage of the FBR, the body attempts to quarantine the external object in a fibrous capsule. Monocytes, macrophages and foreign body giant cells adhere at the implant interface. Fibrous tissue will form, surrounding these white blood cells, and walling the implant from the host tissue. Outside this fibrous tissue, a highly vascularized granulation tissue is formed.

### 7.3. Implant Design for Optimal Biocompatibility and Biostability

If biocompatibility issues are not addressed in a design, the device will have a disturbed functionality during or after the foreign body reaction, according to several factors:

- Grafting factors: depending on the implant surface morphology and properties, the FBR might be mild or strongly pronounced, resulting in a smooth integration in the local tissue or in the presence of a thick fibrous encapsulation or even a chronic infection.
- Implant fabrication factors: proper implant design should prevent adverse effects such as corrosion caused by biological fluids penetrating into the device, or release of toxic implant material in the organism.
- Sensor biofouling factors: due to accumulation of biological material on the sensor surface, the sensor performance might be impaired.

#### 7.3.1. Grafting Factors

Upon implantation, the FBR should be mild, resulting in a smooth integration of the implant in the local tissue. The formation of a thick fibrous encapsulation during the FBR should be avoided in case the implant is carrying biosensors, since for most biosensors, this barrier will often result in an incorrect or a delayed sensor read-out. Finally, biofilm formation at the implant surface should be prevented at all means. Biofilm formation is the accumulation of bacteria at the implant surface, and is a cause of chronic infection. Due to the slimy substance used by the bacteria to encapsulate themselves, the removal of the bacterial colonies by antibiotics is almost impossible. Once a biofilm is formed, removal of the implant is often the only solution to overcome infection, hence prevention of biofilm formation by a fast and smooth integration of the implant into the local tissue is crucial.

Grafting solutions aim to improve cell adhesion, promote vascularization and reduce inflammatory stimuli. A first solution consists of the use of engineered implant coating or implant surface treatments. The material morphology, porosity and mechanical properties can influence cell signalling, organization, proliferation and migration [211]. For example, at cellular dimensions, molecular diffusion is dictated by the geometry of the cellular interface with the material, the neighbouring cells, and the surrounding aqueous micro-environment. At larger scales (>10^2^ μm), material architecture determines bulk mechanical properties, cell migration, and nutrient and waste exchange [211]. In this respect, Sharkawy *et al.* [212] have shown that porous coatings of polyvinyl alcohol can promote higher vascular density, tissue in-growth and faster response to analytes compared to smooth surfaces. After this initial work, several groups have started to employ nanostructured shells for implantable sensors, using material like titanium [213] and silicon [214].

Other approaches, which promote blood vessel formation and reduce fibrosis, are based on porous poly-L-lactic acid coating [215], hydrogels [216–218], and coatings containing certain growth factors [219]. In order to reduce the inflammation and biofilm risk upon implantation, the use of drug releasing coatings can be considered, such as a coating of hydrogel loaded with dexamethasone, a strong anti-inflammatory agent [220,221].

#### 7.3.2. Implant Fabrication Factors

Active implants typically consist of microchips, a battery, and other components, which are (at least partially) made from non-biocompatible materials. The diffusion of such materials into the body tissue should be avoided by all means. Furthermore, biofluids should not penetrate into an active implant: biofluids will corrode the device and might promote leaching of toxic compounds into the body. As a consequence, a bi-directional diffusion barrier is essential: body and implant must be isolated from each other. Moreover, biocompatible materials must be used for the fabrication of this diffusion barrier and the packaging of the total device [222,223]. The most conventional device package consists of a titanium box, equipped with feed-throughs for sensors. The titanium package of a pace-maker is well known, having dedicated ceramic/polymer-based feed-throughs for the leads. Titanium is biocompatible and the box is sufficiently thick to prevent all diffusion. Disadvantages of this package is its size and the rigidity of the box, which is most often in strong contrast with the local tissue and hence not ideal with respect to the resulting FBR. Smaller implant packages make use of encapsulation by ceramic materials and/or polymers.

In order to reduce implant dimensions, ceramic or polymer films can be used. They have the advantage of being compatible with wafer-level processing and to conform to any shape. Thin films can be divided into organic or inorganic.

Most popular organic compounds for packaging include SU-8, epoxies, silicones, polymides, polyurethanes and parylene-C. Attention should be paid when using these materials for long-term implants, since some of these materials can be affected by moisture penetration and biofluid etching. Among these compounds, parylene-C is very promising due to its excellent biocompatibility and step coverage, and its high resistance to moisture penetration [224–226]. A drawback of parylene-C is its limited resistance to heat. Polymide is very attractive as a flexible substrate for implantable electrodes [227]. Due to some remaining moisture permeability, additional measures should be taken to protect the implant from moisture penetration.

Among inorganic films, silicon nitride is the most widely employed for moisture protection [228]. Metal films can be also used for hermetic packaging, when deposited onto another polymeric film which provides electrical isolation. Iridium oxide, titanium, titanium nitride, tantalum and tantalum nitride can be biocompatible if proper deposition conditions are respected [229]. Noble metals like platinum and gold present high corrosion resistance and biocompatibility, and represent excellent candidates for the microfabrication and the connection of the subcomponents of an implantable device. An example is the fabrication of a Utah Electrode Array as reported by Topper *et al.* [230]. Disadvantages of these metals are their ductility and the cost.

#### 7.3.3. Biofouling Factors

Biofouling is the accumulation of biological material on the device surface. In contrast to biofilms, which consist of bacteria, this biologic material is not causing an infection. This aggregation of cells, macromolecules and small molecules on the biosensor membrane has been extensively reported as detrimental for the sensor function [231–234]. Biofouling often prevents diffusion of the analyte or adherence of the analyte on the sensor surface.

Several strategies have been employed to reduce biofouling, most of them rely on the use of dedicated membranes and coatings. An extensive review about this topic can be found in reference [235]. Below we will briefly describe two techniques for reducing biofouling: hydrogel overlays and Nafion coatings.

Hydrogel overlays, mainly made with poly(hydroxyethylmethacrylate) or poly(ethylene glycol), present a hydrophilic interface which can favor diffusion of water-soluble analytes. Diffusion rate is controlled by changing the crosslinking density of the gel. A drawback of this approach is their poor adhesion to the substrate, and a poor mechanical stability during the implant.

Nafion is a commercial, perfluorinated polymer that gained a lot of popularity as a coating for glucose sensors. It is characterized by chemical inertness and both hydrophobic and hydrophilic properties. An additional reason for its extensive use, is its easy application by dip-coating.

**Figure 16.**
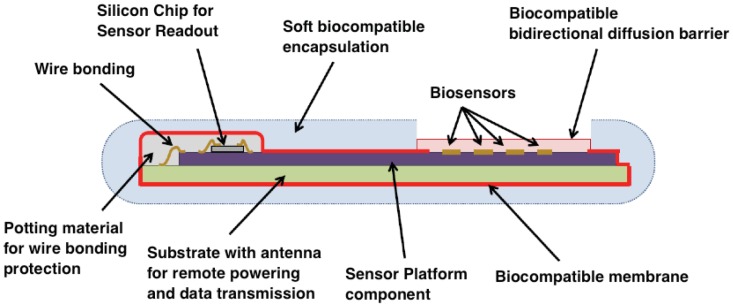
A reliable biocompatible packaging.

The natural response of the host to the introduction of exogenous material is called the *‘foreign body response’* (FBR). It basically consists in wound healing, combined with an attempt to digest followed by walling of the implant by fibrous tissue. Promoting smooth wound healing and minimizing the extent of the FBR is one of the major goals to accomplish when optimizing for biocompatibility of an implanted device. The selection of all components and raw materials for device fabrication (Figure 16) will influence its biocompatibility, as well as the integration techniques used to realize the final device.

Hence, biocompatibility is concept of primary importance, starting from the design of implantable biosensors, including all aspects of their fabrication, until the follow up of the implantation. This implies that in order to realize successful implantable devices, extensive communication between experts of different disciplines is essential starting from the most early development phase.

## 8. Security and Privacy

In order to complete a fully integrated sensing system, an end-to-end data acquisition system must be designed. This ranges from the control and calibration of the actual sensors, to analog-digital conversion of the data, to signal processing, local data storage, source coding, channel coding, transmission, and finally reliable, secure storage in back-end data-bases, and each of these needs to consider potential failure modes as well as threat models, while satisfying constraints on power and cost. Many of these techniques are well known and have been studied extensively in the field of sensor networks [236]. However, security and privacy are critical design aspects in biosensors due to their presence within and interactions with the human body.

Data security is typically characterized by three dimensions: (1) confidentiality; (2) integrity; and (3) availability [237]. Each of these presents unusual challenges in biosensors. As a foundational principle, all primary and derivative biometric data, including biosensor data originating from an individual should be considered private and owned by the user. Accordingly, data should be confidential and encrypted to avoid observation by unauthorized parties. If this privacy is violated, the data can be used in illegal or unethical ways, for example, insurance fraud or discrimination. The integrity of the data along with its origin and timestamp are also critical to preserve and protect. Data from a biosensor can be used to control drug-delivery systems [238] or electrical therapies [239], thus it is crucial to protect the data from tampering. Finally, the availability of the device should be protected such that it can perform its intended function, even in an environment remote from an administrator. Although many techniques can be applied from recent work in lightweight security and privacy for embedded devices such as wireless sensor nodes, RFID tags, and smart cards, biosensors present several different challenges.

### 8.1. Special Features for Biosensors

For this paper, we restrict ourselves to the class of biosensors, which actually perform as a lab on chip, conducting a small experiment at the molecular level on the sensor. Recent examples of this type include implantable/injectable subcutaneous devices that are remotely powered by a bandage-like patch which also provides a data-link to a higher level wearable device, possibly a body-area-network (BAN), and eventually higher levels of health information systems [164]. A related class of devices is that of low-cost disposable biosensors for detecting infectious disease or critical levels of glucose and lactate in a battlefield or other trauma situation [240]. However the constraints and threat models are significantly different since the device is not implanted. Data security and privacy issues for these types of sensors differ significantly from those of higher data-rate sensors such as brain [241] or intraocular [242] implants. For example:

– A particular sensor may take several minutes to complete its task, and only deliver several bytes of data. Thus, the information from the biosensor can have a very high value per bit. This presents challenges for cryptography since the space of possible data values is quite small.
– Energy issues range from remote-powered devices with very close power sources and energy harvesting to non-rechargeable batteries. Energy can be optimized by exploiting the very slow data rates, using sub-threshold voltage circuits or other techniques, which trade off performance for efficiency. However energy starvation is another threat vector [239].
– Error protection must be considered in addition to malicious tampering of data. Again, the very small amount of data means there is very little inherent redundancy, so error correction codes should be used. Integrity verification provides some built-in detection and correction of single bit errors but parity bits should be added to account for multiple bit errors [243].
– Threat scenarios are varied. Although eavesdropping on a properly operating sensor may be unlikely due to the very short wireless channel (a few mm for a sub-cutaneous sensor), impersonation of both reader and patch are plausible concerns. For example, the patch of an unconscious patient can be removed and replaced by another patch. Similarly, a rogue sensor can upload fraudulent data to a trusted patch.

Since biosensors create data, which is eventually used for larger scale health information systems and health care decisions, there are several additional considerations.

– Liability in health care may warrant the use of audit techniques. Sensor data can be used to help determine liability in case of malpractice or other wrong-doing [244].
– Security considers the impact of a malicious adversary. Safety issues related to operator error, design error or other unintentional malfunctions are probably a more likely concern and need to be balanced in the design.
– Emergency access to the sensor device may justify over-riding the security mechanism. A medical first-responder may need to disable the security of the device to gain access to life-critical data or to enable/disable a therapy (e.g., defibrillator) [239].
– Medical devices are regulated in most nations (e.g., FDA in the US) so it is important to detect data from unapproved devices.
– Medical devices are expensive and a prime target for counterfeiters. Cryptography techniques such as physical unclonable functions (PUFs) and watermarks can be used to identify genuine devices and the data they produce.
– Calibration, test and control of the sensor require a secure downlink. Threats arise due to deliberate miscalibration, which can result in erroneous measurements. Unsecured test and debug access is notorious for providing back-door access for attackers.

### 8.2. Alternative Architectures

Recently, a number of solutions have been presented to the general problem of implantable medical devices (IMDs) that include biosensors. In [245], a lightweight wireless protocol for IMDs is presented, leveraging existing technologies and emphasizing low-energy computation.

In [239], both eavesdropping and power-depletion attacks are considered. To address power-depletion, a novel but simple approach is developed that uses RFID-style remote powering until the authentication process is completed, at which point, more general access to the implanted device and battery are enabled.

The choice of encryption scheme should consider the nature of the data as well as the device constraints. In [246], the lightweight stream-cipher Hummingbird is implemented in hardware to encrypt data from a glucose sensor. In [242], block ciphers are explored for use in IMD security, although the target device is an ocular sensor with high data-rates.

In [247], a novel device worn around the neck provides a radio-level shield against unauthorized eavesdropping, jamming and access. This novel approach avoids modification of existing implanted devices (which would otherwise require new surgery to replace the device) and leverages the physical proximity of the shield, although it is external to the body.

In a similar approach, Sorber [248] proposes an amulet to provide broad protection for wearable medical devices. Cornelius [249] presents an intriguing approach based on accelerometers to detect if two devices are on the same body and thus experiencing similar movement. This can help prevent the challenges of the near-far problem where an eavesdropping or unauthorized device could actually be closer to the sensor than the intended receiver (e.g., in a crowded elevator). Document [250] proposes the use of keyed ultra-wideband communication in medical devices to allow low probability of intercept and even detection. UWB is also expected to provide better resistance to physical layer identification techniques based on RF signatures [251]. Biosensors present a set of new and diverse challenges for security and privacy and a unique combination of constraints. A recent review of these and the more general challenges of securing implantable medical devices can be found in [252].

## 9. Future Perspectives

**Figure 17.**
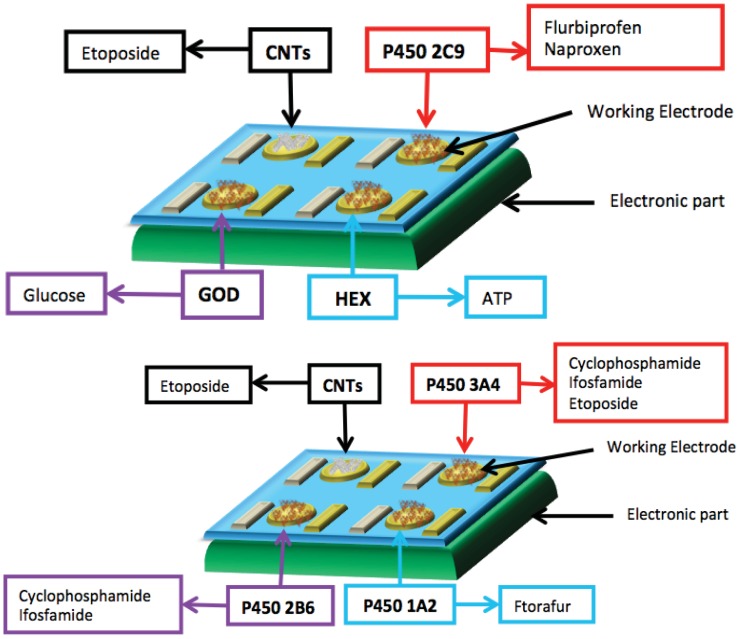
Two possible integrated biochip platforms.

Fully integrated biochip platforms for advanced healthcare are possible by considering all of the levels considered in this paper, from electro-chemistry, to biocompatibility, to sensor electronics, and eventually the security and privacy of the acquired data. Figure 17 shows a platform that can host two different micro-array biochips for two different applications. In the first case (on the right), several sensors having targets on drug compounds for anti-cancer treatments are on board. By choosing the right isoform of the probe enzymes from the family of cytochromes P450 we can develop a fully integrated platform for point-of-care application on chemotherapy patients. The considered drugs in the example are cyclophosphamide, etoposide, ftorafur, and torsemide, all compounds used in chemotherapy cocktails [253]. Of course, the possibility to monitor the amount of the anti-cancer compounds in the patients' blood provides the tuning and personalization of the therapy on patient's metabolism.

The example reported on the left of Figure 17 is instead related to the monitoring of both curing compounds and disease biomarkers. In fact, this configuration of the platform provides the measure of patient's metabolism biomarkers (glucose and ATP) as well as the sensing of anti-cancer (etoposide and cyclophosphamide) or anti-inflammatory (naproxen) drugs. As we have seen in Section 3, several other metabolites as target of the platform might be considered just by choosing the right probe enzymes to be immobilized onto the sensing micro-array.

**Figure 18.**
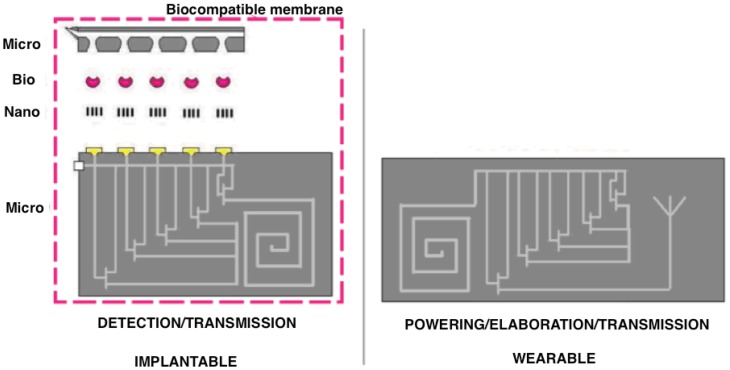
Two systems: the implanted and wearable ones.

**Figure 19.**
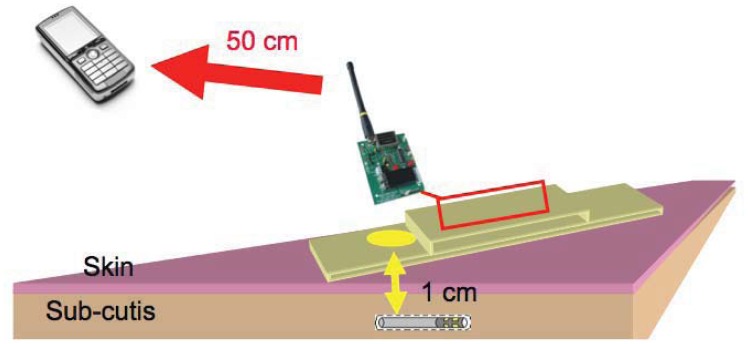
The i-needle under the skin, the remote-powering patch, and the mobile phone for connection to a geographical network.

Platforms like that shown in Figure 17 require special co-design [254] of the supporting electronics circuitry for autonomous operations, as described in Section 5. So, a fully-integrated platform requires both an implanted device as well as a supporting external station for data receiving and power transmission, as schematically shown in Figure 18. The implantable device needs to have on board not only the detection front-end (Section 5) but also nano-materials to improve the sensors performance (Section 4), biomaterials providing the right specificity (Section 3), may be a bit of microfluidics and a biocompatible package (Section 7) is necessary as well as the remote powering system (Section 8). The supporting external station has to be located not far away for constraints coming from the physics of remote powering. A good solution that has been discussed in Section 8 is to have an electronic patch as light as possible just on top of the skin over the implant region, as shown in Figure 19. In that manner, a mobile phone network assures geographical connection to the hospitals or the healthcare services.

**Figure 20.**
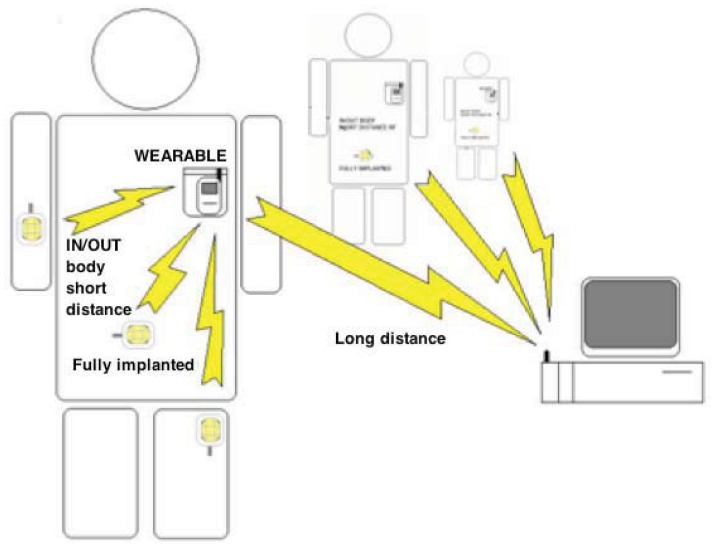
Applications to remote monitoring of human health.

Gathering mobile network also means opening to remote control of patients in parallel from the hospital to elsewhere (Figure 20). So, full management of patients at home or during their normal life activities is then possible with such kind remote monitoring biochip platforms in order to assure advanced healthcare services for fast intervention in case of fatal events, personalized therapy, continuous support to chronic and elderly people, *etc*.

## 10. Conclusions

This paper has presented an overview of the key issues to be addressed in developing fully integrated platforms for innovative biosensors with applications in advanced healthcare. We have seen how to improve the device sensitivity and to decrease the limit of detection by using different nanostructured materials (Section 2). We have discussed how to select the right bioreceptors and how to immobilize such bioreceptors (Section 3) on the sensing surfaces. We have considered the integration of bio and nano-materials onto micro-arrays (Section 4) by using the most advanced technology for heterogeneous systems. We have sketched appropriate CMOS circuits to provide the electronics front-end (Section 5) and the required remote powering (Section 6) to develop highly integrated implantable devices. We have highlighted the relevant problems of device biocompatibility (Section 7) and data security (Section 8) in order to show the future perspective of these fully integrated systems for advanced healthcare (Section 9). Full integration means solving all the problems concurrently and in common between the different sub-systems, and solving the new problems that usually arise from the integration of parts that are not automatically compatible—systems integration. Typical examples are the case of different bioprobes requiring different electrochemical queries facing a single common electronics front-end or that of the opposite needs in remote powering: definitely small receiving antennas in the implant to reduce patient pain and large receiving antennas to provide enough energy to the implants. So, substantial ongoing efforts are required and expected from the worldwide research and development community to succeed in fully operating autonomous and low-cost integrated platforms for advanced healthcare. Despite these challenges, we expect that in a five-ten year horizon we can anticipate reliable technology for the remote telemetry of the human metabolism.
